# ‘You Should Be Yourself’—Secondary Students’ Descriptions of Social Gender Demands

**DOI:** 10.3390/children12040502

**Published:** 2025-04-14

**Authors:** Karin Bergman Rimbe, Helena Blomberg, Magnus L. Elfström, Sylvia Olsson, Gunnel Östlund

**Affiliations:** 1 School of Education, Culture and Communication, Mälardalen University, 721 23 Västerås, Sweden; 2 School of Health, Care and Social Welfare, Mälardalen University, 721 23 Västerås, Sweden; helena.blomberg@mdu.se (H.B.); magnus.elfstrom@mdu.se (M.L.E.); sylvia.olsson@mdu.se (S.O.); gunnel.ostlund@mdu.se (G.Ö.)

**Keywords:** mental wellbeing, emotional wellbeing, gendered performativity, gendered wellbeing, adolescents

## Abstract

**Background/Objectives:** Swedish schools are mandated to counteract gender norms that restrict students’ life opportunities. School personnel also bear the responsibility of fostering students’ democratic responsibilities and healthy behaviors, which is crucial not only for their mental wellbeing but also for their academic performance, as stressed by the European Commission. **Aim:** The purpose of the present study is to explore adolescents’ performativity of gender when discussing social barriers to mental and emotional wellbeing. **Methods:** Fifty adolescents were interviewed in small gender-divided groups, and the transcribed text was analyzed using thematic analysis. Theoretically, interactionist perspective and gender analytic discourses are applied. **Results:** Emotional barriers to mental wellbeing were identified based on too cogent gender norms. Boys describe challenging each other and the environment by using a social facade that includes “stoneface” and harsh language, seldom showing sadness, even among close friends. The girls’ facade includes maintaining a “happy face” and trying to be attractive. Both genders underline the need for belonging, and most of them fear social exclusion from peers. According to the interviewees, it is socially acceptable for girls to display most feelings, even mental difficulties such as anxiety or phobia, but among boys, gender norms still hinder them from showing emotional vulnerabilities such as sadness and risking exclusion. **Conclusions:** Young people’s emotional wellbeing needs to be further developed and included in the curriculum. It is time for adults to focus on boys’ sadness and depressive emotions, as well as girls’ aggressiveness and frankness rather than their appearance, to push the river of equality forward.

## 1. Introduction

This article explores adolescents’ mental [1] and emotional wellbeing [2,3] with a focus on their perceived gender situations in the school context. In the twenty-first century, adolescent mental health needs have been accentuated, and gender differences have been identified in research [2,3,4,5,6,7,8,9,10]. Despite significant advancements over the past century in women’s rights and queer rights and evolving social standards toward equality, youths still predominantly adhere to binary gender roles, which are reinforced by their school environments [11,12,13,14]. Students who belong to a minority group or have disabilities [1] are at a greater risk of discrimination, and less belonging in general contributes to less mental wellbeing [14]. Neurodiversity is more commonly recognized today, and research based on students’ experiences shows that their difficulties in learning situations often cause school absenteeism in both boys and girls [15].

Teachers try to form a positive school culture with functional classroom practice and to give a sense of community within each school [12,13]. However, research shows that teachers often view and treat boys and girls differently, which contributes to maintaining a hierarchical system in which gender differences are reinforced in the classroom [16]. Gender differences in school appear to be established early. By middle school, teachers perceive boys and girls as occupying ‘different arenas’, with girls already reporting lower levels of school wellbeing than boys. Teachers often view girls as ambitious and independent in their schoolwork, while boys are seen as requiring more support, reminders, and encouragement [16]. Despite outperforming boys at all educational levels, many girls do not perceive STEM (science, technology, engineering, and mathematics) subjects as suitable for them, except for girls with migrant backgrounds [14].

Teachers also tend to respond to violations between students based on the perceived severity of the violation and the victim’s reaction. The more vulnerable and weaker the victim is perceived to be, the more reaction and support they receive from teachers [17]. Similarly, violations against girls are more frequently noticed because they are more likely to report incidents to teachers or other adults. Not all boys are violent—some are well-behaved in school—and not all girls are compliant; some are disruptive and struggle academically [6].

Girls’ emotional wellbeing decreases during adolescence in contrast to that of boys, who seem to be more content with their life situation [3]. Previous research has shown that girls tend to internalize life challenges, sometimes resulting in depression or anxiety, while boys are more likely to externalize them through behavioral reactions [7] or substance use [8]. Boys in upper-level lower secondary schools believe that belonging to a group is crucial for standing out in school [9]. These social demands for group homogeneity seem to be overarching general learning needs, with boys underperforming more than girls do [15] and, to a greater extent, contributing to a negative school climate through verbal abuse or threats of violence [12,13]. Both boys and girls perceive boys as mischievous and poorly behaved in school, yet also strong and resilient, both mentally and physically. Any behavior that deviates from gender norms is policed by both genders [18].

Girls are less likely to offend others than boys are, but those who ‘fight back’ in offending situations face double punishment from peers and adults, as they violate the female norm of non-violence [19]. Boys, on the other hand, often feel more distant from anti-bullying groups and report receiving less support from teachers when they are victimized [10,12,13]. Boys are also more likely than girls to attribute the cause of the violation to the victim’s own behavior or characteristics [20]. Violations can occur within friendships and peer groups, making them harder to detect and address for both the victim and those around them. However, violations within friendships can cause significant suffering for the victim [21,22]. 

Boys offend other students more often than girls and are also more exposed to systematic verbal maltreatment. Boys’ violations are, to a greater extent, physical, while those committed by girls tend to be verbal, although verbal violations are also common among boys. The verbal abuse in the boys’ group—so-called harsh language—can be seen as part of the male norm, as boys should ‘tolerate’ more, according to both them and the adult world [23,24,25,26,27,28,29,30]. More than boys, girls attribute peer violations to an individual’s negative appearance and clothing, reflecting the gender norm that girls should be attractive [24]. In schools that have major problems with violations, girls are also exposed to physical violations, albeit to a lesser degree than boys [27,28,29,30,31].

This study attempts to fill the gap in previous research and primarily involves presenting the students’ own voices regarding their views on wellbeing. This research can therefore contribute to broadening the perspective on the gendered wellbeing paradox that affects women and men around the world [32]. Sweden’s fifth-place ranking on the World Economic Forum’s list of countries with the smallest gender gaps [33] makes it even more interesting to study gender issues among Swedish youth, including whether schooling has been able to form them into more equalized youngsters.

### 1.1. Theoretical Framework

This article adopts an interactionist perspective [34] and a social constructionist approach [35], focusing on how gender is created and performed in relation to others through discursive understandings of social reality. Gendered discourses, which define what is considered male and female, contribute to and shape the gender identities that are possible in different contexts. Gender discourse analysis comes from poststructuralist feminist theories that emphasize how gender is socially constructed. Therefore, gender is not a natural or biological feature of individuals but is created through continuous actions and interpretations in everyday life [33,34]. The interactionist perspective posits that our identity is formed through interactions with others, with socially and culturally accepted roles in each context playing a crucial role. The context and the individuals present determine the roles we assign or attribute to ourselves [33,34,35].

These roles come with associated obligations and expectations, meaning that once a person assumes a role, they are also expected to behave in a manner that is socially and culturally appropriate. Gendered discourses can thus reveal both what is associated with and expected of young people’s different roles or genders and their power relations within a school context [33,34]. One of Butler’s [34] central concepts is performativity, which posits that people continuously perform, present, and produce their perceived sex and gender. In Butler’s view, performativity explains how gender identity shapes people and is linked to ideas about sex assignment, bodies, and sexuality. However, constantly repeating gender norms can lead to new and unexpected ways of expressing gender [33]. Gender is a performance created through the repeated actions of individuals, making gender roles appear stable [33,34]. This performativity is also embedded in language [33,34].

The formation and (re)production of identity in social interaction can be illustrated using theatrical metaphors: people perform in front of others according to social expectations, with this performance taking place on the ‘front stage’. It is ‘backstage’ that people can relax and prepare for their social roles [35]. Identity formation occurs not only in face-to-face interactions but also in virtual forums such as Snapchat, TikTok, and Instagram, where responses from others may be delayed in time and space [36]. Digital arenas for identity formation and interaction with friends and followers are part of the social reality to which young people relate, providing opportunities to create an online self [36,37].

A facade is the habitual and regular pattern of behavior that is perceived and observed in a person during a performance. During a performance, an individual can consciously or unconsciously use a personal facade to control the impression they wish others to have of them in the current situation. The personal facade includes the overall impression of the person and the details associated with them, such as social position, clothing, gender, age, appearance, ethnicity, posture, speech patterns, facial expressions, and gestures [35].

### 1.2. Aim

The school environment both shapes and challenges these gender identities. It is through interactions with adults and peers that young people position themselves in relation to gender identities. The norms encountered and adhered to in school, along with the values imparted at home and the treatment of young individuals by both institutions, significantly shape young people’s self-presentations and interactions with others [38]. The present study aims to provide a deeper understanding of what mental and emotional wellbeing entails from adolescents’ perspective.

The purpose of the study is to explore adolescents’ performativity of gender when discussing social barriers to mental and emotional wellbeing.

Research questions:

How do adolescent students experience perceived social demands in a school context?

How do they uphold or oppose prevailing gender differences?

## 2. Material and Methods

The design of the study was abductive, and qualitative data were collected through small group interviews. Giving participants enough space to remain the main actors in the interview situation includes asking them to expand on their answers and interpretations [39]. Group interviews were chosen to get the conversation moving more easily and to support the adolescents’ free speech in regard to their thoughts and views.

### 2.1. Participants and Group Interviews

Sex-separated interviews were conducted with 50 students, comprising 27 girls and 23 boys, from three upper-level lower secondary schools in the same city (Table S1). The students attended three public schools situated in the same town but in different places, which made it possible to include students from different socioeconomic backgrounds: one school in the city center, one in the suburbs, and another in the countryside. The students volunteered to participate in the interviews after receiving information about the study. To contribute to an easy situation for the participants, everyone could choose with whom they wanted to be interviewed or to be interviewed alone. Nine participants (seven girls and two boys) chose to be interviewed alone.

In consultation with school leaders and teachers, the time and place of the interviews were determined, and the interviewer met the students at each school. The interviews lasted approximately one hour, with a variation of 30 min. The decision to conduct sex-separated interviews was made by the municipality, which had girls as its priority as they are an exposed group when it comes to psychological ill-health. Therefore, the first year of the project focused solely on girls.

In the second year, boys were included based on students’ feedback regarding perceived unfair conditions in the project. This time, only two schools were included when we conducted interviews. We decided to stop the data collection for two reasons: The summer holidays were approaching, and we also had a comparable amount of data from girls and boys.

### 2.2. The Interviews and the Interviewer

The interviewer asked the participants to discuss their thoughts on emotional wellbeing and mental health, specifically what made them feel good or bad at school. As the interviewees were divided based on sex, the gender issue was a continuous topic in relation to mental health and emotional wellbeing at school. The interviewer, an experienced researcher with a background in social work with youth and families (GÖ), came to explore the topic of gender since this made interviewees more engaged in discussions on emotional wellbeing and mental health. The focus on the mental health of adolescent girls was determined in the preplanned grant committee and by the research group and was approved by the ethics committee in the first of two steps, the first year concerning girls and the second boys. Since both boys and girls reacted to the lack of equity in the preplanned data collection through interviews in the project, boys were also included, as mentioned above. The interviewer, being a woman and therefore potentially biased toward the girls, ensured neutral and inclusive interviews for the boys, mostly drawing on her experience working with boys in sensitive situations but also as an adult who did not work at the school, which allowed the boys and girls to perceive a certain freedom to speak freely during the interviews.

### 2.3. The Empirical Material and the Analysis

First, the text was read, and the sound file was listened to. The two were then checked against each other. Then, preliminary themes were marked throughout the text by three researchers (KBR, HB, GÖ), two of whom focused on the data transcriptions analyzing both girls’ and boys’ experiences of the school context. The third researcher focused on when girls and boys told of any experience in relation to any gender. After a general analysis of the material, our determination was that both boys and girls became more engaged when talking about gender. It seemed as if it was possible to talk about social demands rather than the difficult terms of mental health and emotional wellbeing. The boys’ and girls’ descriptions and discussions of emotional experiences in school situations often related to gender roles and social demands based on gender, although this did not mean that everyone talked about the opposite gender. Upon rereading the interviews, we found that the interviewees referred to gender issues in the conversations when talking about wellbeing. The small group interviews were more about following the participants’ voices concerning how they experienced their school situation and finding what aspects engaged them.

The analysis of the interviews was based on Clarke and Braun’s [40,41] six-stage Tmodel of reflective thematic analysis. First, the interviews were transcribed and read in their entirety to familiarize ourselves with the material. In the second step, initial codes that were found in all interviews were generated, marked, and compiled into a document. In the third step, these codes were sorted into themes. During steps two and three, the codes and themes were tested, and in the fourth step, the material was read several times again to ensure nothing was missing. In the fifth step, the different themes were named. In this step, we returned to the research questions to ensure that they were answered. In the final step, the results section was constructed based on this. In the work, both individual and collaborative reflection were important. Two of the researchers primarily processed the material into these themes, with the first author being the main responsible. In its final form, it was read and processed by all three researchers. In addition, all three had access to the transcribed material, which was important as it enabled the collaboration required to conduct thematic analysis [39,40,41,42].

An abductive approach was used to adhere to the theoretical approach in the last part of the thematic analysis [41]. The integration of two theoretical perspectives [34,35] was useful in capturing the main themes of the young people’s descriptions.

### 2.4. Ethical Considerations

Ethical considerations have been continuously addressed throughout the research process. Students under the age of 15 who participated in the interviews had their guardians’ written approval to take part in the research, while those who had already turned 15 were allowed to decide for themselves whether to participate in accordance with the research ethics code in Sweden. The guardians were informed about the project at school by the research group [42]. Participation in the interviews was voluntary, and during class, the researchers informed the students of their rights, including the right to choose not to answer specific questions or to withdraw from the interviews at any time without their school situation being affected.

### 2.5. Validity and Reliability

To ensure the study’s validity and reliability, several steps were taken. The study’s design and the data collection methods were discussed and scrutinized by the researchers, but the data material had already been collected before half of the research group was included. Two members of the first research group are still part of the group. Thus, the present analysis of data was conducted stepwise and was reread by new research colleagues close in time. The researchers read, reread, and discussed the interview material before the thematic results were finalized. The data material has also been compared with new interviews with adolescents using a similar methodology [43].

The study design and research question were approved by the Swedish Ethical Review Authority. The first part of the study included only girls in the interviews, and the changes to the research design to include boys were also approved by the Swedish Ethical Review Authority.

## 3. Results

The analysis identified two key themes illustrating how adolescents describe and perform gender. The first theme, social cohesion or risk exclusion, addresses how boys and girls risk social exclusion and experience pressure to conform to gender behavior based on being on a stage where viewers can always see them. The second theme, social facades—happy face and stoneface, illustrates how (young) people of today continuously focus on self-presentation. This is also based on viewers’ perceptions IRL (in real life) or on the internet.

The study’s key finding is that youngsters feel they need to live up to certain gender norms and that they identify this regarding not only their own sex but also the opposite sex. One of the biggest fears among both boys and girls is exclusion, and doing gender is one way to ensure one is part of the group cohesion.

### 3.1. Social Cohesion or Risk Exclusion

Most adolescent girls and boys search for friendship and belonging in groups, which seems to be a basic condition for their emotional wellbeing. The social group dynamics at school, in which a main concern is being excluded, directly relate to the students’ emotional wellbeing, irrespective of gender:

*G9a: “Not many people want to be with me, and that’s because they push me away. I don’t have that big social circle like everyone else. I don’t know why that is. It’s spread that I’m not like everyone else and I’m wrong according to everyone else.”*

*G9b: “I really don’t understand why it would be wrong to not be like everyone else; you should be yourself.”*

(Girl Interview 9)

The quotes from these girls (Girl Interview 9) concern one girl’s possible social exclusion due to not being like the others (G9a) and her friend’s encouragement to be herself (G9b).

Confirmation from others is crucial for most adolescents, irrespective of gender. The significance of peer relationships is repeatedly emphasized, with the absence of social connections linked to feelings of alienation and a reluctance to attend school. One participant described this as follows:

*“Being frozen out and not having anyone to be with or sit with means that you don’t want to go to school and that you feel extra pressure for how the school day should develop. Learning things under too much pressure and stress doesn’t work at all.”*

(Girl Interview 12)

The experienced threat of possible exclusion makes you want to stay home instead of going to school and also makes learning impossible.

*“It’s friends—if you become popular, if you make a lot of friends—then you’ll feel fine every day in school. Then you know you have friends who want to be with you at lunch breaks and stuff, whereas if you don’t have any friends, then you just feel bad about coming here and watching everyone else having fun and having people to be with.”*

(Boy Interview 2)

The boy quoted above underlined the importance of having friends; otherwise, you’ll feel bad about coming to school (B2). Adolescents feel pressure to act normally, often based on gender demands. As a girl, you are expected to get high grades; if you do not, you are not acting girlish enough, and you might feel different and like you are not part of “that big social circle”:

*G11b: “You have to have much better grades than the boys, anyway!”*

*G11a: “Lots of people assume that girls have better grades than boys, and if you don’t, you’ll be snubbed too. It creates stress if you’re not part of that big social circle, and then you become ostracized, and you start to feel bad about… that.”*

(Girl Interview 11)

Social exclusion and academic pressure not only impact emotional wellbeing but also impair learning, as stress and fear of exclusion create an environment that is inconducive to academic engagement. There are different forms of establishing and maintaining a social connection to one’s gender group; while the girls mention grades and being successful in school, boys talk about the need to talk like a boy. Using harsh language is perceived to be preparation for adult and professional life, as this will be expected by future male workmates:

*“That’s what it is. I think like this: In working life, craftsman professions and so on, then it’s very harsh language, and girls shouldn’t, girls don’t belong there. Of course, it should decrease, I think it should decrease, but as it is now, you need to show that you come from such an upbringing to be accepted. You can’t act like an office rat. You should show, it’s a lot like this, you should show that you’re from the countryside. […] Well, it’s like this: a girl and a guy in the class, and I’m telling them both the same joke, and I know that both might not, let’s say they don’t appreciate the joke, but the guy just ignores it, and maybe he worries more about the girl, how she feels after having heard that joke.”*

(Boy Interview 1)

The boy quoted above demonstrated that he thinks girls are different from boys and that boys must be more sensitive toward girls. In the interview, the boys say that the harshness within the boy group should not be given much attention, as it is considered normal boys’ talk. On the other hand, when girls express a harsh view, it might be dismissed with remarks like, ‘*Are you on your period or something?*’ (Girl Interview 11). However, one of the boys finds this bullying talk among friends childish:

*“I have a pretty strange relationship because, in my friend group, we all bully each other, which is kind of strange and, like, I would prefer it if it wasn’t like that, although that’s how it is.*

*Interviewer: But can’t you talk about it?*

*No. Everyone in my group is really ridiculous like that. It’s a little childish, and it’s kind of hard, but you have to live with it, so I don’t really care anymore about that or about their childishness, although I wish they would be a little more mature. […] It’s kind of common to be called gay [for example]. It’s like walking around and being called by your name.”*

(Boy Interview 8)

Both boys and girls frequently encounter sexist comments about their appearance or the use of derogatory terms referring to, for instance, being a whore or a gay person. However, the word gay is not used against boys known to be homosexual; calling a boy gay rather serves to describe him as an outsider in relation to the typical boy group. Trans girls are also excluded from ‘*that tough thing*’ and are not included in the boy group either, while trans boys are not mentioned at all, with trans girls only mentioned in one interview (Boy Interview 7). Boys might also use bullying ‘*to keep someone in line*’ (Boy Interview 2). This description included a boy who had lost a close relative, after which his ‘*friends*’ teased him and tried to force him to act according to his gender, but they failed, and he chose to change school. According to the interviewees, bullying is part of the general social habits that both genders seem to use to react to differences:

*“I’ve seen a lot of girls who bully others too. I’ve seen a girl who wasn’t wearing makeup, and a lot of girls walked past her and had makeup on and said, ‘Look, look, you’re so disgusting, you don’t have any makeup on, you’re not pretty, you’re not, you don’t smell of perfume, you don’t have nice clothes.’ And for girls, uh, it’s like when girls are bullied, it’s worse than a guy being bullied; girls are more sensitive than guys.”*

(Boy Interview 5)

The girl quoted below claimed that she took care of herself before adapting to the gender group, which excluded her from some of the girls. She said she had learned from her older sisters how to improve her wellbeing but had to face the other girls, who were provoked by her independent style:

*“I’ve had a fight with a girl here at school before, and it was like this: […] that if boys fight, all the teachers go in and separate them, but there were teachers standing and listening to what she said to me, but no one went in […] because there were so many bad things and she lied so I would look bad, but none of the teachers went in anyway because it wasn’t considered as bad as boys fighting.”*

(Girl Interview 14)

### 3.2. Social Facades—Happy Face and Stoneface

This theme explores how students handle feelings that are not accepted by their gender group. Two major distinctions are identified: girls are expected to be more in touch with their emotions but still put on a ‘*happy face*’, while boys tend to adopt a ‘*stoneface*’ in interactions with others, almost regardless of who the peer company consists of:

*“I think it’s harder to tell guys how you feel than girls because you might not even notice that the person’s feeling bad since they’re good at hiding it. Then it all depends, […] even if you’re good enough friends with certain people, they […] may seem completely normal the whole time you’ve known them. But they might trust you enough to tell you, and you still don’t notice a difference at school because they’re kind of so good at hiding it.”*

(Boy Interview 3)

The quoted boy (B3) also argued that boys stop crying before puberty, while girls continue to cry. According to the interviewees, when boys display signs of sadness or distress, they are often ignored by those around them; for instance, when a boy started crying during a language lesson, everyone, including the girls, averted their eyes to avoid seeing him cry (Boy Interview 9). However, some boys also react against these gender norms:

*“As a guy, you’ve been taught that you should kind of stand up and be a man and not show so many emotions, although I think it’s important, of course, that you should. It’s always good to say how you feel and to show emotions; it’s important—I think so. But you usually don’t notice it [feelings]. You might recognize that the person is down since he walks alone a lot or kind of looks down and has this kind of sad face; then it’s clear that this person’s very sad.”*

(Boy Interview 8)

Another boy is sure that using a stoneface is childish and wrong. He also has experience in supportive conversations.

B6: *“But it’s wrong, the wrong idea [not showing emotions]; everyone has feelings, so sometimes you might feel like crying. You’re allowed to cry, but a lot of people see guys as emotionless and more, like, angry.”*

(Boy Interview 6)

Boys are often expected to hide sadness, but one participant expressed hope that upper secondary school would allow him to express his emotions openly. The digital world is also part of adolescent life; girls especially talk about how the digital world forces them to put on a happy face no matter how they feel inside. According to them, you have to put on a happy face on social media irrespective of how you feel:

*“You can sit and hide so much behind the screen. You can sit and cry and feel so bad, but you can post a picture on Instagram and say you’re feeling great; you might take a picture when you’re really happy, or you look happy. Although you’re actually broken—that is, broken inside—you can sit and sort of hold the phone, sit and cry and be completely destroyed. […]. So, you can hide a lot behind social media.”*

(Girl Interview 3)

According to both girls and boys, feeling bad is something that girls use as an excuse to get out of lessons, claiming, for instance, ‘*I have anxiety and need to leave the classroom*’. Using mental health causes as an excuse for taking a break is accepted by others; although it is sometimes questioned, it is seen as a way to get out of class. Girls also talk about others who post pictures when hurting themselves, which is also often questioned:

*“I also believe that maybe they [people who self-harm] post [pictures] because they want help, but they don’t get it because some people may think like ‘you’re just an attention seeker’. And I can also agree that it’s kind of annoying because they want attention. You just [want to say] go and talk to someone so you can get out of this [negative situation], but it could also be that they post it because they need help!”*

(Girl Interview 13)

Questioning girls’ emotional expressions and behavior as a possible plea for external validation is not unusual. Carefully applied makeup is a part of the social facade that girls must maintain to fit in. Wearing makeup is also an important part of not appearing too ‘boyish’, according to the girls, because without this, they risk being harassed and teased:

*“Because boys want […] boys want to be boys and girls shouldn’t be like that [like them]. Girls have to look nice, and boys have to behave in a certain way, and girls […] are less worthy and have to [look nice]; otherwise, they get comments and things like that from the boys.”*

(Girl Interview 3)

Accordingly, keeping up a nice appearance as a girl is a demand both from boys and girls. Both genders describe being expected to have a well-trained body and to look “nice” in order to please and to fit in with the group. The boys also describe how demands about having the right body shape, clothing style, and looks affect their emotional wellbeing and self-confidence:

*“Being unsure about your own body, not wanting to show it because you might get bullied, like getting picked on and stuff. And then it gets so overwhelming that it just makes you feel bad, so you don’t want to; you just lose all your self-confidence. That might be why you want to fit in with your body, fit in with things like eye color, fit in with clothing style, and all that.”*

(Boy Interview 2)

The experienced insecurity regarding physical appearance and the intense pressure to conform to masculinity standards, including the fear of being bullied, further emphasizes the need to be accepted by the boys’ group. The excerpt above from Interview 2 highlights the influence of media and peer pressure on body image and behavior. The emphasis on both physical appearance and wearing the right outfit is tied to the masculinity norms and one’s social worth on the front stage. The influence of what others wear in school and in popular culture shapes both boys’ and girls’ perceptions of ideal physical attributes and clothing styles:

*“Like, I was bullied; there are some students who say to each other, ‘Look, you don’t have Adidas pants, those aren’t Adidas, you’re not rich, you’re poor.’ […] And then you don’t dare wear those clothes like you’re going to feel ashamed, uh, you’re just going to feel bad, I can’t afford to buy as much as he can […] so you feel like you don’t have the same value. […] A friend of mine got a sweater as a gift, and people at school said it was fake, and then he went home and threw that sweater away.”*

(Boy Interview 5)

Body and appearance are closely related to both boys’ and girls’ wellbeing, as gender norms and social demands are delivered directly by one’s schoolmates—especially if you do not adapt to them.

## 4. Discussion

The aim of this study was to explore the performativity of gender when discussing social barriers to mental and emotional wellbeing in the school context. The study’s contribution is that it underlines the gendered experiences of social demands that are made visible through peers’ actions and reactions in the school context and on the internet. During the interviews, both girls and boys talked about how they had to perform according to social demands based on gender performativity [37]; otherwise, they risk exclusion from the group and might lose their sense of belonging [8]. Most young people fear exclusion if they are not able to meet the prevailing gender standards. Every school day, you have to reinforce the right self-presentation on the front stage [35]. Girls articulate the struggle to present a happy face, indicating a gendered expectation to curate an aesthetically pleasing and emotionally detached persona online and, of course, IRL. As a girl, you are also supposed to have higher grades than boys. According to the interviewed boys, they are expected to have muscles, the right outfit that does not reveal sadness or vulnerability, and they have to present a ‘*stoneface*’ on the front stage. Emotionality does not fit in at all for boys, except for aggressiveness [36]. This hegemonic masculinity [43,44,45] includes emotional control and physical dominance and not showing vulnerability, especially sadness.

However, one of the boys acknowledges the importance of being emotionally up-front—‘*it’s always good to say how you feel*’—revealing an awareness of the value of emotional openness despite conflicting societal expectations. This dual perspective suggests an internal negotiation between traditional gender norms and new, emerging views that promote emotional transparency [36]. The interviewees underscore the persistence of traditional masculinity norms that emphasize emotional suppression as a marker of strength and maturity. Such stereotypes not only constrain boys’ emotional freedom but also perpetuate a damaging cycle in which their emotional needs are suppressed, leading to potential long-term consequences for their mental health and emotional wellbeing. The interviewees perceive the potential emotional isolation and reduced access to social support networks if they do not adapt to the gender demands, underscoring the importance of addressing emotional literacy and communication skills, particularly among boys, in order to challenge restrictive gender norms and promote emotional wellbeing.

Boys are socialized into homosociality in which it is important to be and do like the others, with harsh langauge setting the framework for how boys should act, using language in which homosexual and trans youth are excluded. This ‘boy talk’ is both offensive and includes harassment, which boys dismiss as something that is part of being a boy [12,13]—although one of the interviewed boys in the present study does not agree about using this childish, harsh language. The excerpts shed light on gendered expectations and hierarchies that reinforce inequalities between boys and girls. The interviewees highlight how boys are socialized to adhere to specific masculine behaviors (‘boys want to be boys’), while girls are expected to conform to ideals of appearance and submissiveness (‘girls have to look nice’). This reinforces gendered stereotypes that position girls as less worthy and more vulnerable to criticism from and control by boys [36]. The statement also reveals how deviations from these gendered expectations result in social sanctions, such as comments and judgment. This social dynamic suggests that gender norms not only dictate behavior but also sustain power imbalances and reinforce gender hierarchies through social policing [34].

The boys interviewed in the present study are aware that girls do not accept the hard boy language; this becomes another way of doing gender, with the boys being those who ‘*endure*’ and the girls being those who are supposed to be ‘*sensitive*’. The violations and harassment that the students describe seem to be derived from what they perceive as deviant appearance and manner, i.e., breaking the gender norms [28]. Even if one changes the way one acts, as one of the girls did when she chose not to adapt to the group, gender is performed nonetheless, as the other girls reacted to her different way of acting [34].

Only boys mention having been a part of what is called bullying and repeated harassment, but only one boy admits having had the role of bully. Previous research [31] also found that few people would admit to bullying. The girls in this study do not mention having been bullies but agree with the boys that both sexes are involved in several situations that could be classified as violations and repeated harassment; but to call it bullying would be to assume a—probably undesirable—victim role. The girls, on the other hand, feel pressured to follow the requirements involving their appearance or be outcasts; to avoid this, they prepare themselves for the front stage [35].

### 4.1. Keep Up the Facade

Of all young people, girls are generally described as more emotional. However, in this study, the boys emphasize in their interviews that they differ from the girls in this way. Girls who show sadness and mental difficulties are ‘accused’ by both girls and boys of doing this to get attention and receive benefits such as getting out of lessons. Girls’ bad feelings are ignored and relativized in this way. When girls express their vulnerabilities, there is also an accusation that these players have a front stage that does not correspond to their backstage [35]. The boys in the study also offer the example that girls hold onto things while boys let go much more quickly of the life difficulties they experience, a gender difference that is also reinforced in research on adults [32]. However, the supposed difference between boys and girls also further reinforces the notion that boys should not care about emotional situations, including sadness and depression, while girls are considered much more sensitive, which is another way of establishing the difference between the genders [34,36,37].

How school is managed is another area where students take opposing positions and shape different competing discourses [37]. The girls in this study experience pressure to be successful, as they are expected to get higher grades than the boys. Girls’ interest in school becomes a gender difference maintained by students [34,35]. The reproduction of gender differences can also be understood in terms of expectations and obligations set by adults both at school and at home.

Boys are not allowed to show that they feel psychologically bad in any way. According to both the boys and girls in this study, boys should maintain a stoneface and not show sadness and/or depressive emotions, even to their best friends. The only acceptable way to exhibit a sad mood that the boys mentioned in the interviews is through aggressive behavior. However, boys who show that they feel psychologically troubled through aggressive behavior risk being met with further harsh language by the boy group and, in the long run, being left outside the group, even though the boys rarely talk about exclusion and do not expect that any of the boys would suffer serious mental distress in such a sequence of events.

The findings reveal that teenage boys’ mental and emotional wellbeing is deeply intertwined with their ability to maintain group belonging when handling school situations, not least when their self-confidence is at stake and/or they risk losing face. Accordingly, the social group dynamics and conformity demands related to masculinity norms and social belonging have a major impact on boys’ wellbeing. The teenage boys interviewed in this study talked about how they adapted to conformity demands and perform boyhood in relation to the group by having a trendy appearance, using masculine talk at least among the boys, and suppressing emotional signs of weakness, especially in the school context [26,34,38,45].

If someone infringes on the boys maintaining their stoneface, both boys and girls become incredibly uncomfortable. This reflects the gendered discourse regarding boys who do not show emotions [34,37] and the notion that students must clearly keep the front stage and backstage separate [35]. Boys are not allowed to show on their faces what goes on behind the scenes “backstage”. They are forced to maintain a social facade, which is shared by several boys in the interviews because part of the expectation around being a boy is to not show sad or depressive emotions. Some girls, however, offer examples of having to pretend to feel fine in order to maintain the social facade of being successful on the front stage [35].

In an international setting, these findings echo previous research, and not only in the Swedish context. Rigid gender norms and their influence on educational achievements are found in several studies [2,5,6,7,9]. In previous studies, girls who break the gender norms are therefore seen as failing twice, both academically and in regard to conforming to the norms for their sex [16,20]. These girls are not visible in the current study, but it has been mentioned several times that school failure is not acceptable for girls in any way. Other notable similarities with international studies are the rigid norms boys must conform to and the importance of belonging, especially among boys [22,23,29,32]. Girls’ norm conformity is visible in international studies, just as it is in this one. One notable aspect of international studies is that gender norms affect adolescents’ academic pathways [20,22]. The findings in this study do not indicate equal conditions among Swedish secondary students compared to other countries. Although several of the interviewees were critical of the social demands they experienced, they felt they had to conform to gender roles yet wanted the situation to be different.

### 4.2. What Does the Adult World Need to See, Understand, and Reflect On?

The present study echoes previous knowledge about boys’ supposed lack of interest in learning and a repetition of the male norm of not showing emotion and instead using harsh language [4,5,6,7,14,16,18,19,22,23,26,28,29,30]. It is easy to sympathize with teenage boys who act on the front stage [35], which does not allow them to reveal emotions such as sadness and a depressive mood but only reinforces that boys are allowed to act out through aggressiveness.

Moreover, gender norms expect girls to be interested in getting high grades and maintaining a perfect appearance. The interviewees here describe how the pressure comes from the outside in regard to high grades, appearance, and not showing all types of emotions, depending on gender. The awareness of this pressure from the outside in young people’s talk shows us that gender norms are continuously changing in line with how, according to Butler [34], they are constructed and acted upon on both the front stage and backstage [35].

The findings in this study could be used by educators and policymakers to understand and then take into consideration how young people perceive norms and stress factors, particularly in school. This could help create a more inclusive environment and support for both boys and girls in school, according to the pressure they experience in their school environment. Educators can consider gender pressure in their everyday teaching, and policymakers can do so when addressing topics like grading systems and national tests.

### 4.3. Future Studies

All adolescents in this study subject themselves to a stereotypical heterosexual identity. LGTBQIA+ (lesbian, gay, trans, bisexual, queer, intersex, asexual, plus) are mentioned as “the other”; also, no experiences involving a migrant background are mentioned in the interviews. Future studies would benefit from investigating these groups of adolescents and their experiences. It would also be of great interest for observations to be carried out in schools, as well as interviews with educators and other school personnel. Additionally, a study including both younger and older students could significantly broaden our understanding of the gender development topic, potentially teaching us more about how classroom situations can be used for reflecting on gender demands and how they can be counteracted.

In this study, clear gender differences are shown and the results themselves reinforce these differences. Other questions and a different focus might have produced different results. However, this study can serve well as an introduction to how young people’s mental and emotional wellbeing is partly neglected by adults. To advance gender equality, adults should prioritize addressing boys’ sadness and depression as well as encouraging girls’ assertiveness and frankness rather than focusing on appearances. Hence, it’s time to tell every person, irrespective of their age, ‘*You should be yourself*’, as one of the interviewed girls suggested to her friend.

### 4.4. Methodological Considerations

This research project started as a study of girls’ experiences of and knowledge about poor mental health. However, the students who were invited to participate, as well as some of the school personnel, reacted to the project idea as being unequal with regard to gender, which allowed us to include boys in the second project year. Based on the students’ engagement and experiences of subject gender and emotional wellbeing in the school context, this area was more relevant to study. Naturally, this study has its limitations. The sample size of students (N = 50) from one municipality entails limitations of generalizability. However, as a sample of Swedish youth, it is highly interesting since there is no reason to assume these adolescents are unique in their perceived gender norms compared to their peers both in Sweden and abroad.

## Data Availability

The interview data are unavailable due to privacy and ethical restrictions.

## Supplementary Materials

The following supporting information can be downloaded at: https://www.mdpi.com/article/10.3390/children12040502/s1, Table S1: the number of adolescents participating in the two gender divided phases of the research project. The interviews were conducted in Sweden with girls (2018) and with boys (2019).
