# Supplementary material for: ‘You Should Be Yourself’—Secondary Students’ Descriptions of Social Gender Demands
Source: Children (Basel). 2025 Apr 14;12(4):502. doi: 10.3390/children12040502 (PMC12025717; doi:10.3390/children12040502)
Supplement: Supplementary file 1 [file children-12-00502-s001.zip › children-3551015-supplementary.pdf]

Table S1, the number of adolescents participating in the two gender divided phases of the research project. The interviews were conducted in Sweden with girls (2018) and with boys (2019).

Gender	School location and number of interviews (number of participants)		
	Suburban	Countryside	City center
Girls	5 (7)	6 (11)	4 (9)
Boys	5 (13)	4 (10)	
Total	10 (20)	10 (21)	4 (9)
